# Functional, neuroplastic and biomechanical changes induced by early Hand-Arm Bimanual Intensive Therapy Including Lower Extremities (e-HABIT-ILE) in pre-school children with unilateral cerebral palsy: study protocol of a randomized control trial

**DOI:** 10.1186/s12883-020-01705-4

**Published:** 2020-04-14

**Authors:** R. Araneda, S. V. Sizonenko, C. J. Newman, M. Dinomais, G. Le Gal, E. Nowak, A. Guzzetta, I. Riquelme, S. Brochard, Y. Bleyenheuft, Early HABIT-ILE group, Julie Paradis, Daniela Ebner-Karestinos, Geoffroy Saussez, Anne Klöcker, Rodolphe Bailly, Sandra Bouvier, Josselin Demas

**Affiliations:** 1 Institute of Neuroscience, Université catholique de Louvain, Avenue Mounier 53 box B1.53.04, 1200 Brussels, Belgium; 2 Division of Child Development and Growth, Department of Pediatrics, University of Geneva, Geneva, Switzerland; 3 Paediatric Neurology and Neurorehabilitation Unit, University Hospital of Lausanne, Lausanne, Switzerland; 4 CHU Angers, Département de Médecine Physique et de Réadaptions, CHU Angers-Capucins, Angers, France; 5 Université d’Angers, Laboratoire Angevin de Recherche en Ingénierie des Systèmes, (LARIS) – EA7315, Angers, France; 6 University Hospital of Brest, Brest, France; 7 INSERM CIC 1412, Brest, France; 8 Department of Developmental Neuroscience, IRCCS Fondazione Stella Maris, Pisa, Italy; 9 Department of Clinical and Experimental Medicine, University of Pisa, Pisa, Italy; 10 Department of Nursing and Physiotherapy and Research Institute on Health Sciences (UINICS-Idisba), University of the Balearic Islands, Palma de Mallorca, Spain; 11 Western Britany University, Brest, France; 12 INSERM UMR 1101, LaTIM, Brest, France; 13 Pediatric rehabilitation department, Fondation Ildys, Brest, France

**Keywords:** Cerebral palsy, Intensive training, Toddlers, Randomized controlled trial, Functional changes, Neuroplasticity, Biomechanical changes

## Abstract

**Background:**

Cerebral palsy (CP) causes motor, cognitive and sensory impairment at different extents. Many recent rehabilitation developments (therapies) have focused solely on the upper extremities (UE), although the lower extremities (LE) are commonly affected. Hand-arm Bimanual Intensive Therapy Including Lower Extremities (HABIT-ILE) applies the concepts of motor skill learning and intensive training to both the UE and LE. It involves constant stimulation of the UE and LE, for several hours each day over a 2-week period. The effects of HABIT-ILE have never been evaluated in a large sample of young children. Furthermore, understanding of functional, neuroplastic and biomechanical changes in infants with CP is lacking. The aim of this study is to carry out a multi-center randomized controlled trial (RCT) to evaluate the effects of HABIT-ILE in pre-school children with unilateral CP on functional, neuroplastic and biomechanical parameters.

**Methods:**

This multi-center, 3-country study will include 50 pre-school children with CP aged 1–4 years. The RCT will compare the effect of 50 h (two weeks) of HABIT-ILE versus usual motor activity, including regular rehabilitation. HABIT-ILE will be delivered in a day-camp setting, with structured activities and functional tasks that will be continuously progressed in terms of difficulty. Assessments will be performed at 3 intervals: baseline (T0), two weeks later and 3 months later. Primary outcomes will be the Assisting Hand Assessment; secondary outcomes include the Melbourne Assessment-2, executive function assessments, questionnaires ACTIVLIM-CP, Pediatric Evaluation of Disability Inventory, Young Children’s Participation and Environment Measure, Measure of the Process of Care, Canadian Occupational Performance Measure, as well as neuroimaging and kinematics measures.

**Discussion:**

We expect that HABIT-ILE will induce functional, neuroplastic and biomechanical changes as a result of the intense, activity-based rehabilitation process and these changes will impact the whole developmental curve of each child, improving functional ability, activity and participation in the short-, mid- and long-term.

**Name of the registry:** Changes Induced by Early HABIT-ILE in Pre-school Children With Uni- and Bilateral Cerebral Palsy (EarlyHABIT-ILE).

**Trial registration:**

Trial registration number: NCT04020354-Registration date on the International Clinical Trials Registry Platform (ICTRP): November 20th, 2018; Registration date on NIH Clinical Trials Registry: July 16th, 2019.

## Background

Cerebral palsy (CP) affects between 2 to 3.6 out of 1000 live births [1]. It causes abnormal patterns of movement and posture, and cognitive and sensory function may also be impaired [2, 3]. These impairments result from structural abnormalities in the brain that occur at different periods of development [4, 5]. The timing, extent and location of these abnormalities determines subsequent whole-brain functioning [4, 5]. Symptoms generally become apparent before 12 months of age and the diagnosis is usually confirmed between the ages of 12 to 24 months [6, 7]. Many rehabilitation approaches have been developed over the last 15 years to reduce motor dysfunction and its impact on activity and participation [7, 8]. Intensive, activity-based, goal-directed interventions have been shown to effectively improve motor function in school-age children with CP [9], yet the majority of brain growth and development occurs before this during the first 2 years of life. Despite this, few studies have evaluated the effectiveness of intensive rehabilitation and the mechanisms underlying the response to therapy in pre-school children [7].

Animal models of cerebral palsy have shown that impairments due to perinatal brain injury are mainly secondary to persistent inflammation [10–12]. This inflammation alters neurogenesis, axonal growth and synaptogenesis, as well as causing impairments within the white matter [11, 13]. Studies have shown that these alterations can be partially reversed in animals through early intensive motor skill learning based interventions, if they are provided during the optimal developmental window of opportunity [14–17]. This suggests that an intensive, early intervention, based on motor skill learning could stimulate neuroplastic changes in both the grey and white matter, leading to improvements in functional abilities in children with CP.

Few trials of intensive rehabilitation have been carried out in very young children and, to our knowledge, all have focused on children with unilateral CP [9]. Eliasson and colleagues [18] developed adapted baby-CIMT (Constraint-induced movement therapy) for infants (< 12 months) and several other teams adapted CIMT for children between 2 to 3 years of age [19, 20]. Two other research teams tested the effectiveness of CIMT in children with unilateral CP between the ages of 1 and 6 years [21, 22] and another tested the feasibility of conducting bimanual training in children aged from 2 to 4 years [23]. The results of these studies all showed that intensive rehabilitation is feasible in young children, and provided moderate-level evidence regarding the effectiveness of CIMT or bimanual training in young children with unilateral CP. All the interventions studied focused solely on the upper extremity (UE) [18, 21–23], despite the fact that the lower extremity (LE) is very commonly affected in this population. Hand-arm Bimanual Intensive Therapy Including Lower Extremity (HABIT-ILE) [24] applies the concepts of motor skill learning and intensive training to both the UE and LE. It involves constant stimulation of both the UE and LE through the performance of combined activities for many hours each day over a period of 2 weeks. This rehabilitation approach has been shown to improve motor function of the UE and LE in school-aged children with unilateral [25] and bilateral [26] CP across the 3 domains of the International Classification of Functioning, Disability and Health [27]. However, it has never been tested on a large scale in young infants, and very little is known about functional, neuroplastic and biomechanical changes in infants with unilateral CP. We will address this gap by carrying out a randomized controlled study to evaluate the effects of early HABIT-ILE (e-HABIT-ILE), in pre-school children with unilateral CP on functional, neuroplastic and biomechanical parameters.

### Aims and hypotheses

We designed a randomized controlled trial (RCT) to test our hypotheses immediately following 2 weeks of therapy, and at 3 months. The effect of e-HABIT-ILE on both neuroplasticity and movement characteristics of the UE and LE will only be tested at 3 months.

#### Primary aim

The main objective of this protocol is to evaluate the medium-term (three month) effect of a two-week e-HABIT-ILE program in preschool children (aged from 1 to 4 years). This study will determine the effects of the intervention on the ability to use the more affected hand to carry out bimanual activities (Assisting Hand Assessment (AHA) or Mini-AHA, depending on age) in children with unilateral CP compare the effects of e-HABIT-ILE against usual motor activity including usual rehabilitation (control group).

#### Primary hypothesis

*e-HABIT-ILE will induce greater improvements in bimanual performance in children with unilateral CP than usual motor activity including usual rehabilitation, at three months.*

#### Secondary aims and hypotheses

- Aim: To determine the effectiveness of e-HABIT-ILE on unimanual performance of both the more affected and less affected hands.

*Hypothesis: Melbourne Assessment-2 scores will be higher for the HABIT-ILE group than for the control group.*

- Aim: To determine the effectiveness of e-HABIT-ILE on tactile and pressure pain thresholds for both the more affected and less affected hands.

*Hypothesis: Tactile thresholds will be lower (improved tactile sensation: Semmes-Weinstein Monofilament test) and pressure thresholds will be higher (lower pressure sensitivity: pressure algometry) in the e-HABIT-ILE group than in the control group.*

- Aim: To determine the effectiveness of e-HABIT-ILE on executive function.

*Hypothesis: Scores for working memory and inhibitory control tests will be higher in the e-HABIT-ILE group than in the control group.*

- Aim: To determine the effectiveness of e-HABIT-ILE on global activity performance in activities of daily life.

*Hypothesis: Scores for the ACTIVLIM-CP and the Pediatric Evaluation of Disability Inventory (PEDI) will be higher in the e-HABIT-ILE than in the control group.*

- Aim: To determine the effectiveness of e-HABIT-ILE on social participation.

*Hypothesis: Young Children’s Participation and Environment Measure (YC-PEM) scores will be higher in the e-HABIT-ILE group than in the control group.*

- Aim: To determine the effectiveness of e-HABIT-ILE on the perception of health services.

*Hypothesis: Scores on the Measure of the Process of Care (MPOC-20) will be higher in the e-HABIT-ILE group than in the control group.*

- Aim: To determine the effectiveness of e-HABIT-ILE on the functional goals, as determined by the parents.

*Hypothesis: Improvements on the Canadian Occupational Performance Measure (COPM) will be greater in the e-HABIT-ILE group than in the control group.*

- Aim: To determine the effectiveness of e-HABIT-ILE in producing neuroplastic changes in grey and white matter as well as in brain connectivity.

*Hypothesis: Changes in grey matter, white matter and connectivity as measured by advanced brain imaging techniques, will be greater in the e-HABIT-ILE group than in the control group.*

- Aim: To determine the effectiveness of e-HABIT-ILE to produce changes in LE and UE movement characteristics.

*Hypothesis: Improvements in the kinematic parameters for UE and LE movements will be greater in the e-HABIT-ILE group than in the control group.*

## Methods

### Ethics

Full ethical approval for this study has been obtained in Belgium (B403201316810), France (29BRC19.0050/N2019-A01173–54) and Italy (244/2019). A clear and comprehensive information letter will be given to the parents. If they agree to their child’s participation, parents will be asked to sign a consent form at the moment of inclusion in the study.

### Patient and public involvement

This research was build considering the opinion of an French Association of people with cerebral palsy.

### Study design

**Fig. 1:**
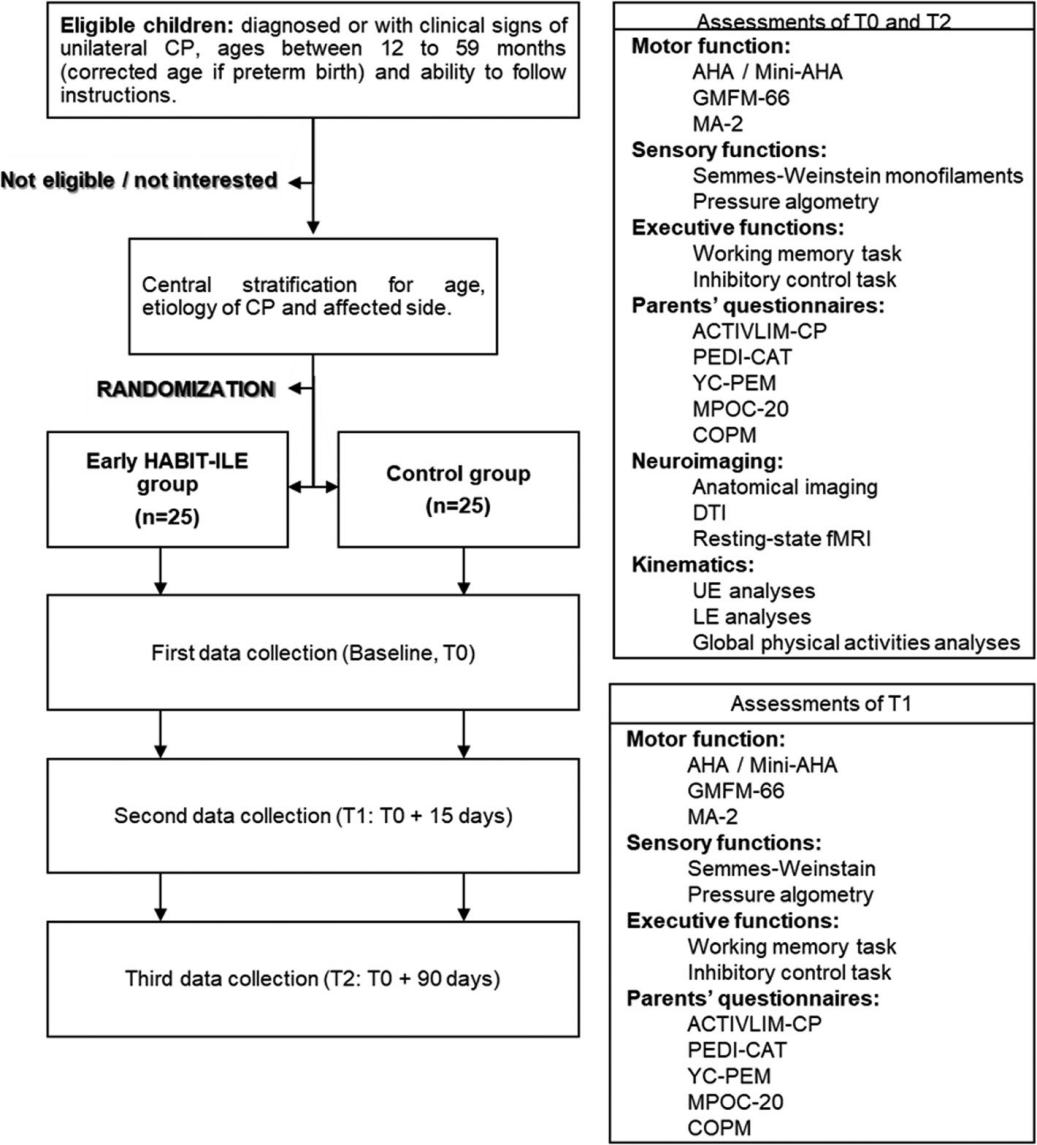
CONSORT Flowchart. RCT, randomized controlled trial; CP, cerebral palsy; GMFCS, gross motor function classification system; AHA, assisting hand assessment; GMFM-66, gross motor function measure (66 items); MA-2, Melbourne assessment 2; PEDI-CAT, pediatric evaluation of disability inventory, computer adaptive test; YC-PEM, young children’s participation and environment measure; MPOC-20, measure of the process of care (20 items); COPM, Canadian occupational performance measure; DTI, diffusion tensor imaging; fMRI, functional magnetic resonance imaging; UE, upper extremities; LE, lower extremities

A multi-center RCT will be implemented across four locations in three different countries: Belgium (Brussels), France (Brest and Angers) and Italy (Pisa). The RCT will involve children with unilateral CP comparing the effect of 2 weeks of e-HABIT-ILE to that of usual motor activity, including usual rehabilitation. Assessments will be performed at 3 intervals: baseline (T0), two weeks after baseline (T1) and 3 months after baseline (T2). The RCT will follow the CONSORT guidelines (See Fig. 1).

### Recruitment

#### Participants

Fifty pre-school children with CP aged between 1 and 4 years will be included. Children will be recruited through the following sites: in Belgium, at the CP center of Saint-Luc University Hospital; in France, at the Brest University Hospital Center and in Italy, at the Centre for Innovative Therapies in CP of the Stella Maris Scientific Institute in Pisa. Also, spontaneous applications from parents of children who are not followed in any of these centers will be considered.

#### Inclusion criteria

Children will be considered for eligibility if they have a diagnosis of unilateral CP, either spastic or dyskinetic, are aged between 12 and 59 months (corrected age if preterm birth), and are capable of following instructions and completing all necessary tests according to their age.

#### Exclusion criteria

Participants will be excluded if they have uncontrolled epilepsy, had botulinum toxin injections or orthopedic surgery within the past 6 months, or if such interventions are planned during the study period, have severe visual or cognitive impairments that could interfere with treatment/testing, have any of the usual contraindications to magnetic resonance imagery (MRI) such as metal implants, or parent(s) are unable/unwilling to provide consent for their child’s participation.

#### Randomization process

Matched randomization will be carried out. Children will be allocated to either the control or the treatment group using a concealed centralized electronic allocation system. The children will be matched for age at entry (1/2/3/4 years) and etiology of CP (brain malformation/ periventricular white matter lesion/ grey matter lesion/) before the baseline assessment and collection of informed consent. In addition, children in each group will be matched for affected side (right/left).

#### Sample size

The sample size was calculated using the results of a previous intensive therapy study performed on infants and young children [19]. That study showed an improvement of 6 AHA-units in the intervention group and of 2 AHA-units in the control group, with an effect size of 1.26 [19]. We also carried out a pilot trial of HABIT-ILE in 10 children with unilateral CP (aged between 1 and 4 years) that yielded a mean increase of 10 ± 6.7 AHA-units at the 3-month follow-up. We therefore expect a minimum effect size of 1 (i.e., an improvement of 1 SD in the HABIT-ILE group compared with the control group) with an α of 0.05 and a 1-ß of 0.9. Therefore, 23 participants are required per group (46 in total). In order to account for potential drop-outs, 50 participants will be included.

### Current status of the protocol

This protocol has already started and is still recruiting.

### Blinding procedure

The main outcomes (AHA or Mini-AHA) will be rated from videos by external accredited/experienced raters unaware of group allocation or assessment interval. Analyses will be performed using study allocation codes. All kinematic analyses, as well as analysis of the neuroimaging data, will also be performed by an experienced rater unaware of group allocation or assessment interval. All data will be anonymized and storage within each site. At the end of the study, these anonymized data will be delivered for analysis.

### Study interventions

#### HABIT-ILE procedure

HABIT-ILE [24–26] is a motor skill learning-based therapy performed in a camp setting, with structured activities of increasing motor difficulty and functional tasks that require the use of both hands whilst performing postural and/or locomotor activity of the LE. We have adapted this program for pre-school children based on the know-how developed in school-age children [24–26] and our pilot study.

HABIT-ILE activities are presented as games and the whole environment is arranged in such a way that children perceive the camp as fun. The activities will be defined by the e-HABIT-ILE supervisors according to the results of the initial assessments (T0) and individualized functional goals that will have been previously defined with the parents (e.g. drinking by him/herself without spilling, holding a book with one hand while turning pages with the other, taking off a t-shirt, etc.). Over the course of the 10 days (2 weeks), the difficulty of each activity will be progressed to ensure that bimanual coordination, postural control and LE function are continuously challenged. Each child will start at a level which he/she can easily achieve. At the lowest level, the more affected UE will be used as a passive stabilizer, with progressive encouragement towards more complex (active) use, through the introduction of games that require more skilled use of the UE. The involvement of the LE in the different activities will also be progressed from: 1) sitting activities e.g. initially on a chair or a mat, potentially with a back support, progressing towards unsupported sitting and sitting on an unstable support, such us on a roller or a ball; 2) transitions from lower to higher postures using the UE for support; 3) static gross motor activities e.g. from standing with UE support to playing in standing without UE support; and 4) dynamic activities e.g. crawling, walking, running or jumping. More skilled/challenging UE activities will be introduced in stable sitting, then the UE activities that have been mastered will be carried out in more challenging LE conditions.

#### Implementation of e-HABIT-ILE

Six to nine children will undergo e-HABIT-ILE simultaneously. Activities will be conducted in both group and individual settings. Each child will have his/her own therapist (PT or OT) as well as a student therapist working with him/her. Two to three therapists experienced in e-HABIT-ILE will supervise the activities to ensure the content of the therapy and structured motor activities are carried out for 80% of the therapy total time. Considering the usual amount of motor activity carried out by pre-school children and infants [28], and allowing for rest periods (e.g. naps), a therapy-time of 5 h per day, (total of 50 h over the study period) was determined to be the best compromise between current scientific evidence on dosage [29, 30] and feasibility in this age group.

#### Control procedure

Children who are randomized to the control group will not undergo any specific activity beyond their routine care (including usual rehabilitation). Toddlers spend around 7.6 h/day executing motor activities [28]. Since a proportion of these activities are performed at home, before or after going to daycare (e.g. dressing bathing, eating), we estimated that around 5 h of daily motor activity are accomplished in daycare [28]. The content of these activities will be documented in a logbook by the caregiver. The amount of activity will be recorded by wrist sensors during 5 days in the treatment and control groups.

#### Data monitoring committee (DMC)

If needed, one researcher of each site will comprise the DMC. They will assess and manage any adverse events throughout the protocol at 6 monthly intervals.

### Outcomes

#### Primary outcome

The primary outcome measure will be the score difference (T2-T0) between the groups (e-HABIT-ILE and control) in the Mini Assisting Hand Assessment [31] (Mini-AHA, for children aged 8 to 18 months old) or the Assisting Hand Assessment [32] (AHA, for children aged 18 months and above). Both tests use Rasch analysis to quantify the assistance provided by the more affected hand to the less affected hand during bimanual activities. These assessments have all been shown to be responsive, reliable and valid for use in children with CP.

### Secondary outcomes

#### Secondary functional assessments (T0, T1 and T2)

The Melbourne Assessment-2 [33] (MA2) will be used to evaluate unilateral upper limb function. It is valid and reliable for the assessment of the quality of upper limb movements in children with neurological conditions.

Tactile threshold will be measured using the Semmes-Weinstein Monofilament Test, which is reliable and reproducible [34, 35]. Pressure threshold will be assessed using an algometer which is well-tolerated by young children [34] and has excellent intra-rater and inter-rater reliability.

Executive functions will be assessed as described by Gottwald et al., (2016) [36], who showed a significant relationship between executive and motor functions in younger children when assessing working memory and inhibitory control.

Global activity performance will be evaluated using the ACTIVLIM-CP [37] which is a reliable and valid questionnaire on activities of daily life involving the UE and LE in children with CP.

Functional skills in daily activities and mobility will be evaluated using The Pediatric Evaluation of Disability Inventory, computer adaptive test (PEDI-CAT) [38]. This four-domains questionnaire has been shown to be sensitive for use in children with CP [39].

Participation will be evaluated using The Young Children’s Participation and Environment Measure [40] (YC-PEM). It has a good intra-rater and inter-rater reliability.

The Measure of the Process of Care [41] (MPOC-20) will be used to measure parent perception of the extent to which health services received by them and their child are family-focused. This self-report questionnaire has been shown to be valid and reliable.

The Canadian Occupational Performance Measure [42] (COPM) will be used to define therapeutic goals and to quantify the child’s performance in relation to these goals, as well as the satisfaction of the parents regarding their achievement. The measure is administered via semi-structured interview and will be performed by a trained examiner.

#### Neuroimaging (T0 and T2)

MRI datasets will be collected in Saint-Luc University Hospital (Brussels), CHRU (Brest) and IRCCS Stella Maris (Pisa), using compatible scanners and standardized acquisition parameters to allow a pooled analysis. All children will undergo an MRI at T0 and T2. Before the scans, all participants will be checked to ensure they do not have any contraindications to MRI. In addition, to avoid undergoing two general anesthesia procedures in a short period, nap- or night-MRIs will be carried out. The infants will undergo a standardized gradual adaptation program so they become accustomed to the MRI environment, including wearing ear-plugs to sleep, bottle feeding just prior to the scan and careful observation of the infant’s sleep routine [43].

Three different sequences will be performed: high-resolution 3D T1-weighted MR images, diffusion tensor imaging and resting-state functional MRI (total time: 20 min). The following analyses will be compared between the control and e-HABIT-ILE groups:

(i) A morphometry analysis focusing on structural neuroplastic changes including cortical thickness, cortical folding and white matter fiber shape will be performed using high-resolution 3D T1-weighted MR images. This approach has recently led to the discovery of biomarkers related to potential correlations between specific tissue lesions and volumetric variations of given cerebral structures, or brain folding [44, 45].

(ii) Changes in fiber quality of the corticospinal tract will be analyzed using diffusion tensor imaging and measures such as fractional anisotropy, main diffusivity and tract volume. The organization of the corticospinal tract and any changes will also be established through fiber tracking [46, 47].

(iii) The topological properties of whole-brain networks will be analyzed using fMRI and graph theory and network analysis. The properties of the motor network will also be calculated [48, 49].

#### Movement parameters (T0 and T2)

The assessments will take place in the 3-dimentional motion laboratories of the consortium (Brussels, Pisa, Brest). These laboratories will use the same standardized acquisition protocol using an optoelectronic system. Children who are able to walk 10 m independently will undergo both LE and UE analyses whilst those who cannot will only undergo UE analysis. The total time to perform both the UE and LE evaluations will be 30 to 45 min.

(i) Gait analyses with synchronized electromyography will be performed at T0 and T2. Sixteen reflective markers will be positioned on anatomical landmarks following the protocol by Davis et al. [50]. Muscle activity will be recorded using a 16-channel electromyography system positioned over the rectus femoris, vastus lateralis, medial hamstrings, tibialis anterior and gastrocnemius-soleus muscles, according to the SENIAM recommendations [51]. Children will walk barefoot, unassisted, along a 10-m-long walkway. At least ten trials will be recorded. Parental encouragement and toys will be used to incite children to walk along the walkway in a straight line. Spatio-temporal, kinematic parameters, and muscle activation indexes will be calculated.

(ii) UE 3-dimentional motion will be performed at T0 and T2. Based on the experience of the Brest team and recent evidence [52] from a study of 10-month old infants, we expect to acquire complete analyses for 60% of the children. During the procedure, a simplified set of upper-limb markers [53] will be positioned on anatomical landmarks over the thorax, shoulder, arm, forearm, and hand. Four electromyographic signals (the long head of the triceps brachii, the short head of the biceps brachii, pronator teres and quadratus) will be analyzed according to the method developed by Sarcher et al. 2016 [54] and the SENIAM guidelines [51]. Children will sit in a standardized seat at a table and will be asked to perform two reach-to-grasp tasks. Spatio-temporal and kinematic parameters will be calculated along with muscle activation indices such as the Arm Profile Score [55] and the EMG-Profile Score for UE motion [56].

(iii) Global physical activity (resting and active states with intensity, posture and gait periods via bar-code parameters) will be measured using wrist sensors [57, 58] (automatic calibration, simple positioning, similar to a watch on each wrist). The main outcome will be the percentage of total time spent in movement (i.e. crawling, walking and running) during either the HABIT-ILE or the control period.

### Statistical analysis

Improvements in AHA (or Mini-AHA) will be compared between study groups using analysis of covariance (ANCOVA) with adjustment for baseline measurements, as recommended by Vickers et al. [59]. Secondary outcome measures will be analyzed using ANCOVA. Non-parametric analyses will be performed whenever ANCOVA assumptions (homoscedasticity and normality) are not met. Age-subgroup (or according to other characteristics) and interaction analyses will be performed for exploratory purposes.

## Discussion

This study protocol describes the background and design of a RCT to demonstrate the changes produced by Early HABIT-ILE in comparison with usual activities. We expect e-HABIT-ILE to induce functional, neuroplastic and biomechanical changes as a result of the intense, activity-based rehabilitation carried out during the stage of major central nervous system development. We expect that these changes will impact the whole developmental curve of each child, improving functional ability, activity and participation in the short-, mid- and long-terms.

The results of this project will be disseminated through peer-reviewed publications and at national and international conferences. If the outcomes demonstrate the effectiveness of e-HABIT-ILE and the dissemination is as large as expected, this should have a world-wide impact on the care of children with CP. The early improvement in the functional capacity of children with CP should reduce the mid- and long-term consequences of the pathology, thus reducing the economic impact of CP on healthcare systems.

## Data Availability

Not Applicable.

## Abbreviations

AHA: Assisting Hand Assessment
ANCOVA: Analysis of covariance
CIMT: Constraint-induced movement therapy
COPM: Canadian Occupational Performance Measure
CP: Cerebral Palsy
DMC: Data Monitoring Committee
GMFM-66: Gross Motor Function Measure
HABIT-ILE: Hand-arm Bimanual Intensive Therapy Including Lower Extremities
LE: Lower Extremities
MA2: Melbourne Assessment-2
MPOC-20: Measure of the Process of Care
PEDI: Pediatric Evaluation of Disability Inventory
RCT: Randomized controlled trials
UE: Upper Extremities
YC-PEM: Young Children’s Participation and Environment Measure

## References

[1] Graham HK, Rosenbaum P, Paneth N, Dan B, Lin JP, Damiano DL (2016). Cerebral palsy. Nat Rev Dis Primers.

[2] Krageloh-Mann I, Cans C (2009). Cerebral palsy update. Brain and Development.

[3] Weierink L, Vermeulen RJ, Boyd RN (2013). Brain structure and executive functions in children with cerebral palsy: a systematic review. Res Dev Disabil.

[4] Chugani HT, Müller R-A, Chugani DC (1996). Functional brain reorganization in children. Brain and Development.

[5] Brizzolara D, Pecini C, Brovedani P, Ferretti G, Cipriani P, Cioni G (2002). Timing and type of congenital brain lesion determine different patterns of language lateralization in hemiplegic children. Neuropsychologia.

[6] Novak I, Morgan C, Adde L, Blackman J, Boyd RN, Brunstrom-Hernandez J (2017). Early, accurate diagnosis and early intervention in cerebral palsy: advances in diagnosis and treatment. JAMA Pediatr.

[7] Reid LB, Rose SE, Boyd RN (2015). Rehabilitation and neuroplasticity in children with unilateral cerebral palsy. Nat Rev Neurol.

[8] Bax M, Goldstein M, Rosenbaum P, Leviton A, Paneth N, Dan B (2005). Proposed definition and classification of cerebral palsy, April 2005. Dev Med Child Neurol.

[9] Novak I, McIntyre S, Morgan C, Campbell L, Dark L, Morton N (2013). A systematic review of interventions for children with cerebral palsy: state of the evidence. Dev Med Child Neurol.

[10] Baud O, Daire JL, Dalmaz Y, Fontaine RH, Krueger RC, Sebag G (2004). Gestational hypoxia induces white matter damage in neonatal rats: a new model of periventricular leukomalacia. Brain Pathol.

[11] Favrais G, van de Looij Y, Fleiss B, Ramanantsoa N, Bonnin P, Stoltenburg-Didinger G (2011). Systemic inflammation disrupts the developmental program of white matter. Ann Neurol.

[12] Van Steenwinckel J, Schang AL, Sigaut S, Chhor V, Degos V, Hagberg H (2014). Brain damage of the preterm infant: new insights into the role of inflammation. Biochem Soc Trans.

[13] Leviton A, Gressens P (2007). Neuronal damage accompanies perinatal white-matter damage. Trends Neurosci.

[14] Friel KM, Williams PT, Serradj N, Chakrabarty S, Martin JH (2014). Activity-Based Therapies for Repair of the Corticospinal System Injured during Development. Front Neurol.

[15] Martin JH, Friel KM, Salimi I, Chakrabarty S (2007). Activity- and use-dependent plasticity of the developing corticospinal system. Neurosci Biobehav Rev.

[16] McKenzie IA, Ohayon D, Li H, de Faria JP, Emery B, Tohyama K (2014). Motor skill learning requires active central myelination. Science..

[17] Xiao L, Ohayon D, McKenzie IA, Sinclair-Wilson A, Wright JL, Fudge AD (2016). Rapid production of new oligodendrocytes is required in the earliest stages of motor-skill learning. Nat Neurosci.

[18] Eliasson AC, Nordstrand L, Ek L, Lennartsson F, Sjostrand L, Tedroff K (2017). The effectiveness of baby-CIMT in infants younger than 12 months with clinical signs of unilateral-cerebral palsy; an explorative study with randomized design. Res Dev Disabil.

[19] Eliasson AC, Shaw K, Berg E, Krumlinde-Sundholm L (2011). An ecological approach of constraint induced movement therapy for 2-3-year-old children: a randomized control trial. Res Dev Disabil.

[20] Nordstrand L, Holmefur M, Kits A, Eliasson AC. Improvements in bimanual hand function after baby-CIMT in two-year old children with unilateral cerebral palsy: A retrospective study. Res Dev Disabil. 2015:41–2. 10.1016/j.ridd.2015.05.003 86–93. Epub 2015/06/24. PubMed PMID: 26100242. 10.1016/j.ridd.2015.05.003 26100242

[21] DeLuca SC, Case-Smith J, Stevenson R, Ramey SL (2012). Constraint-induced movement therapy (CIMT) for young children with cerebral palsy: effects of therapeutic dosage. J Pediatr Rehabil Med.

[22] Taub E, Ramey SL, DeLuca S, Echols K (2004). Efficacy of constraint-induced movement therapy for children with cerebral palsy with asymmetric motor impairment. Pediatrics..

[23] Ferre CL, Brandao MB, Hung YC, Carmel JB, Gordon AM (2015). Feasibility of caregiver-directed home-based hand-arm bimanual intensive training: a brief report. Dev Neurorehabil.

[24] Bleyenheuft Y, Gordon AM (2014). Hand-arm bimanual intensive therapy including lower extremities (HABIT-ILE) for children with cerebral palsy. Phys Occup Ther Pediatr.

[25] Bleyenheuft Y, Arnould C, Brandao MB, Bleyenheuft C, Gordon AM (2015). Hand and arm bimanual intensive therapy including lower extremity (HABIT-ILE) in children with unilateral spastic cerebral palsy: a randomized trial. Neurorehabil Neural Repair.

[26] Bleyenheuft Y, Ebner-Karestinos D, Surana B, Paradis J, Sidiropoulos A, Renders A (2017). Intensive upper- and lower-extremity training for children with bilateral cerebral palsy: a quasi-randomized trial. Dev Med Child Neurol.

[27] World Health O (2007). ICF-CY, international classification of functioning, disability, and health : Children & Youth version.

[28] Van Cauwenberghe E, Gubbels J, De Bourdeaudhuij I, Cardon G (2011). Feasibility and validity of accelerometer measurements to assess physical activity in toddlers. Int J Behav Nutr Phys Act.

[29] Brandao MB, Mancini MC, Ferre CL, Figueiredo PRP, Oliveira RHS, Goncalves SC, et al. Does dosage matter? A pilot study of hand-arm bimanual intensive training (HABIT) dose and dosing schedule in children with unilateral cerebral palsy. Phys Occup Ther Pediatr. 2017:1–16. 10.1080/01942638.2017.1407014 Epub 2017/12/15PubMed PMID: 29240518. 10.1080/01942638.2017.1407014 29240518

[30] Sakzewski L, Provan K, Ziviani J, Boyd RN (2015). Comparison of dosage of intensive upper limb therapy for children with unilateral cerebral palsy: how big should the therapy pill be?. Res Dev Disabil.

[31] Greaves S, Imms C, Dodd K, Krumlinde-Sundholm L (2013). Development of the mini-assisting hand assessment: evidence for content and internal scale validity. Dev Med Child Neurol.

[32] Krumlinde-Sundholm L, Holmefur M, Kottorp A, Eliasson AC (2007). The assisting hand assessment: current evidence of validity, reliability, and responsiveness to change. Dev Med Child Neurol.

[33] Gerber CN, Plebani A, Labruyere R. Translation, reliability, and clinical utility of the Melbourne assessment 2. Disabil Rehabil. 2017:1–9. 10.1080/09638288.2017.1386726 Epub 2017/10/14. PubMed PMID: 29025283. 10.1080/09638288.2017.1386726 29025283

[34] Auld ML, Ware RS, Boyd RN, Moseley GL, Johnston LM (2012). Reproducibility of tactile assessments for children with unilateral cerebral palsy. Phys Occup Ther Pediatr.

[35] Buitenhuis SM, Pondaag W, Wolterbeek R, Malessy MJA (2018). Hand sensibility in healthy young children. Pediatr Neurol.

[36] Gottwald JM, Achermann S, Marciszko C, Lindskog M, Gredeback G (2016). An Embodied Account of Early Executive-Function Development. Psychol Sci.

[37] Bleyenheuft Y, Paradis J, Renders A, Thonnard JL, Arnould C (2017). ACTIVLIM-CP a new Rasch-built measure of global activity performance for children with cerebral palsy. Res Dev Disabil.

[38] Kramer JM, Liljenquist K, Coster WJ (2016). Validity, reliability, and usability of the Pediatric Evaluation of Disability Inventory-Computer Adaptive Test for autism spectrum disorders. Dev Med Child Neurol.

[39] Ko J (2014). Sensitivity to functional improvements of GMFM-88, GMFM-66, and PEDI mobility scores in young children with cerebral palsy. Percept Mot Skills.

[40] Khetani MA, Graham JE, Davies PL, Law MC, Simeonsson RJ (2015). Psychometric properties of the Young Children's Participation and Environment Measure. Arch Phys Med Rehabil.

[41] Siebes RC, Maassen GH, Wijnroks L, Ketelaar M, van Schie PE, Gorter JW (2007). Quality of paediatric rehabilitation from the parent perspective: validation of the short measure of processes of care (MPOC-20) in the Netherlands. Clin Rehabil.

[42] Dedding C, Cardol M, Eyssen IC, Dekker J, Beelen A (2004). Validity of the Canadian occupational performance measure: a client-centred outcome measurement. Clin Rehabil.

[43] Almli CR, Rivkin MJ, McKinstry RC (2007). The NIH MRI study of normal brain development (Objective-2): newborns, infants, toddlers, and preschoolers. Neuroimage..

[44] Dinomais M, Hertz-Pannier L, Groeschel S, Chabrier S, Delion M, Husson B (2015). Long term motor function after neonatal stroke: lesion localization above all. Hum Brain Mapp.

[45] Dinomais M, Hertz-Pannier L, Groeschel S, Delion M, Husson B, Kossorotoff M (2016). Does Contralesional hand function after neonatal stroke only depend on lesion characteristics?. Stroke..

[46] Baek SO, Jang SH, Lee E, Kim S, Hah JO, Park YH (2013). CST recovery in pediatric hemiplegic patients: Diffusion tensor tractography study. Neurosci Lett.

[47] Rose S, Guzzetta A, Pannek K, Boyd R (2011). MRI structural connectivity, disruption of primary sensorimotor pathways, and hand function in cerebral palsy. Brain Connect.

[48] Lee D, Pae C, Lee JD, Park ES, Cho SR, Um MH (2017). Analysis of structure-function network decoupling in the brain systems of spastic diplegic cerebral palsy. Hum Brain Mapp.

[49] Saunders J, Carlson HL, Cortese F, Goodyear BG, Kirton A. Imaging functional motor connectivity in hemiparetic children with perinatal stroke. Hum Brain Mapp. 2018. 10.1002/hbm.24474 Epub 2018/11/18. PubMed PMID: 30447082. 10.1002/hbm.24474 PMC6865539 30447082

[50] Davis RB, Õunpuu S, Tyburski D, Gage JR (1991). A gait analysis data collection and reduction technique. Hum Mov Sci.

[51] Hermens HJ, Freriks B, Disselhorst-Klug C, Rau G (2000). Development of recommendations for SEMG sensors and sensor placement procedures. J Electromyogr Kinesiol.

[52] Ransburg N, Reiser M, Munzert J, Jovanovic B, Schwarzer G (2017). Concurrent anticipation of two object dimensions during grasping in 10-month-old infants: A quantitative analysis. Infant Behav Dev.

[53] Brochard S, Lempereur M, Mao L, Remy-Neris O (2012). The role of the scapulo-thoracic and gleno-humeral joints in upper-limb motion in children with hemiplegic cerebral palsy. Clin Biomech (Bristol, Avon).

[54] Sarcher A, Raison M, Leboeuf F, Perrouin-Verbe B, Brochard S, Gross R (2017). Pathological and physiological muscle co-activation during active elbow extension in children with unilateral cerebral palsy. Clin Neurophysiol.

[55] Jaspers E, Feys H, Bruyninckx H, Klingels K, Molenaers G, Desloovere K (2011). The arm profile score: a new summary index to assess upper limb movement pathology. Gait Posture.

[56] Ropars J, Lempereur M, Vuillerot C, Tiffreau V, Peudenier S, Cuisset JM (2016). Muscle Activation during Gait in Children with Duchenne Muscular Dystrophy. PLoS One.

[57] Bregou Bourgeois A, Mariani B, Aminian K, Zambelli PY, Newman CJ (2014). Spatio-temporal gait analysis in children with cerebral palsy using, foot-worn inertial sensors. Gait Posture.

[58] Newman CJ, Bruchez R, Roches S, Jequier Gygax M, Duc C, Dadashi F (2017). Measuring upper limb function in children with hemiparesis with 3D inertial sensors. Childs Nerv Syst.

[59] Vickers AJ (2001). The use of percentage change from baseline as an outcome in a controlled trial is statistically inefficient: a simulation study. BMC Med Res Methodol.

